# Diverticulitis complicated by pylephlebitis: a case report

**DOI:** 10.1186/1752-1947-5-514

**Published:** 2011-10-10

**Authors:** Mahesh Gajendran, Thiruvengadam Muniraj, Mohamed Yassin

**Affiliations:** 1 University of Pittsburgh Medical Centre, Department of Medicine, 200 Lothrop Street, Pittsburgh, PA 15213, USA; 2 University of Pittsburgh Medical Centre - Mercy, Department of Medicine, 1400 Locust Street, Pittsburgh, PA 15219, USA

## Abstract

**Introduction:**

Pylephlebitis is defined as septic thrombophlebitis of the portal venous system, usually secondary to infection or inflammation in the abdomen. In the current report, we present a case of pylephlebitis that complicated the course of a very common pathology, diverticulitis.

**Case presentation:**

A 62-year-old Caucasian woman with a history of sigmoid diverticulitis presented to our facility with a three-week history of abdominal pain, fevers, chills, loss of appetite and fatigue. Her laboratory test results showed leukocytosis and elevated alkaline phosphatase. A computed tomography scan revealed portal vein thrombosis and a sigmoid diverticulitis with an abscess. Our patient was given pipercillin-tozabactam followed by sigmoid colectomy and loop transverse colostomy. A peritoneal fluid sample culture grew *Escherichia coli*. Our patient had an uneventful post-operative course and the leukocytosis resolved in the next four days. She improved clinically and was discharged home on ertapenem and enoxaparin. A follow-up computed tomography scan two weeks later showed a new pelvic abscess that was drained by a pigtail catheter but there was no change in the portal venous thrombus. A repeat computed tomography scan one month later revealed resolution of the pelvic abscess but persistence of portal vein thrombus, for which enoxaparin was continued.

**Conclusions:**

This is a classic case of pylephlebitis that demonstrates the importance of recognizing that the portal vein thrombus is infected and treating the condition appropriately.

## Introduction

Pylephlebitis is defined as septic thrombophlebitis of the portal venous system, usually secondary to infection or inflammation in the abdomen. The common causes include diverticulitis, appendicitis or cholangitis [1]. Pylephlebitis has to be differentiated from the bland portal vein thrombus. Bland portal vein thrombosis is more common than pylephlebitis and the management is different. Here, we present a case of pylephlebitis that complicated the course of a very common pathology, diverticulitis.

## Case presentation

**Figure 1:**
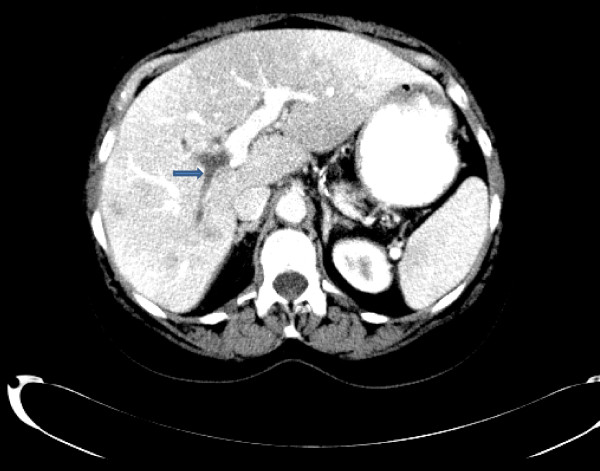
**Computed tomography (CT) scan showing right portal vein thrombosis**.

**Figure 2:**
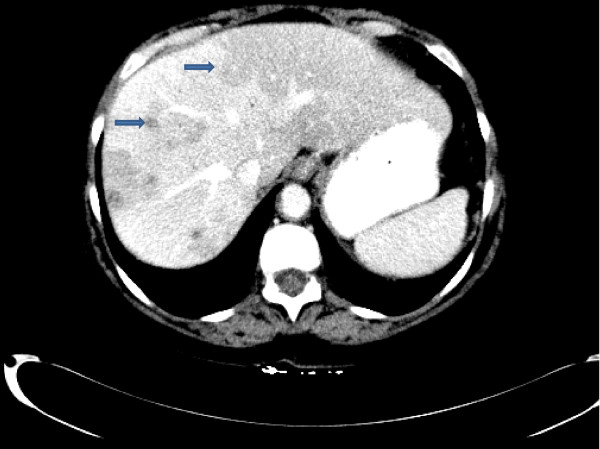
**Computed tomography (CT) scan showing multiple low attenuation liver lesions**.

**Figure 3:**
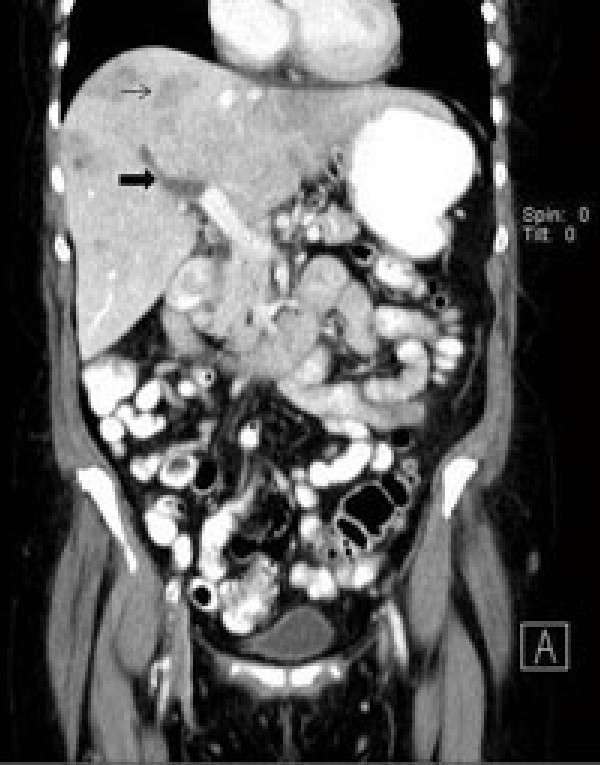
**Computed tomography (CT) scan scout view showing right portal vein thrombus and liver abscess**.

**Figure 4:**
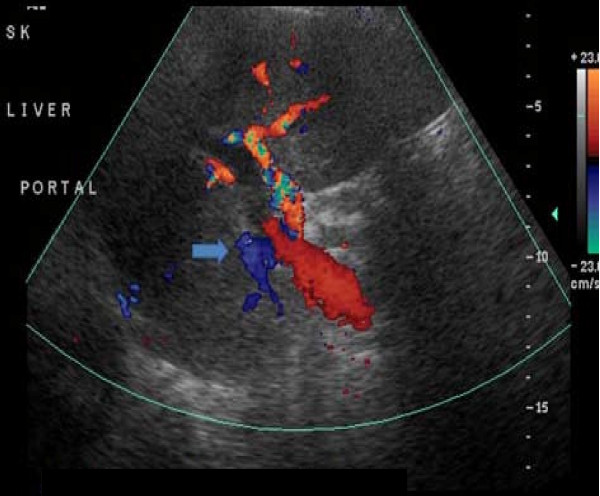
**Color Doppler showing no flow in right portal vein**.

A 62-year-old Caucasian woman with a history of sigmoid diverticulitis (seven months prior to admission) was admitted for three weeks of sharp intermittent left lower quadrant abdominal pain, low-grade fever, chills, loss of appetite and fatigue. She denied diarrhea, bloody stools, nausea, or vomiting. The only abnormal finding on physical examination was tenderness in the left lower quadrant. Her initial laboratory test results showed a white cell count of 17,700 cells/mm^3^, hemoglobin 13.7 gm/dL and elevated alkaline phosphatase two times the normal level. A computed tomography (CT) scan of the abdomen revealed portal vein thrombosis, low attenuation liver lesions (Figures 1, 2, 3) and extensive sigmoid diverticulitis with a 4 × 1.8 cm abscess. This was a new thrombus compared to a previous CT scan, performed two months previously. The color doppler confirmed the presence of portal vein thrombus (Figure 4). An MRI scan of the abdomen did not reveal any additional information. Our patient was given pipercillin-tozabactam followed by exploratory laparotomy, sigmoid colectomy and loop transverse colostomy. An intra-operative ultrasonography of the liver was suggestive of early liver abscesses, but we were not able to aspirate. A peritoneal fluid sample culture grew *Escherichia coli*. Our patient had an uneventful post-operative course and her leukocytosis resolved in the next four days. She improved clinically and was discharged home on ertapenem and enoxaparin. A follow-up CT scan two weeks later showed a new pelvic abscess 7.5 × 6 cm that was drained by a pigtail catheter, but there was no change in the portal venous thrombus. Her hypercoagulable profile was negative. A repeat CT scan one month later revealed resolution of the pelvic abscess but persistence of portal vein thrombus for which enoxaparin was continued.

## Conclusions

Unlike bland portal vein thrombosis, pylephlebitis is more commonly associated with liver abscesses and bacteremia [2]. *Escherichia coli *and *Bacteroides fragilis *are the most common isolates in blood [3]. Doppler ultrasound, CT scanning and MRI scanning of the abdomen has improved the ability to diagnose pylephlebitis [4]. CT scanning demonstrates portal vein thrombus as a non-enhancing, low-density thrombus within the vessel lumen with non-homogeneous enhancement of the hepatic parenchyma [5]. MRI can help to distinguish acute from chronic portal vein thrombosis [6]. Management of pylephlebitis is best achieved by treating the primary source using broad-spectrum intravenous antibiotics and surgical intervention (appendectomy or colectomy with abscess drainage) [1,2]. Early diagnosis and treatment is critical. The role of anticoagulation in the treatment of pylephlebitis is controversial [7].

## Consent

Written informed consent was obtained from the patient for publication of this case report and any accompanying images. A copy of the written consent is available for review by the Editor-in-Chief of this journal.

## Competing interests

The authors declare that they have no competing interests.

## Authors' contributions

All authors equally contributed to the writing of the manuscript. All authors reviewed the final manuscript and approved it for submission.
